# Assessing the impact of hard data patterns on Bayesian Maximum Entropy: a simulation study

**DOI:** 10.1038/s41598-024-70518-z

**Published:** 2024-11-15

**Authors:** Emmanuel Ehnon Gongnet, Codjo Emile Agbangba, Sèdjro A Tranquillin Affossogbe, Romaric Vihotogbé, Romain Glèlè Kakaï

**Affiliations:** 1 Laboratoire de Biomathématiques et d’Estimations Forestières (LABEF), Université d’Abomey-Calavi, 04 BP 1525, Cotonou, Benin; 2 Institut Tchadien de Recherche Agronomique Pour Le Développement (ITRAD), BP 5400, N’Djamena, Chad; 3 Ecole de Foresterie Tropicale, Université Nationale d’Agriculture de Porto-Novo, 01 BP 55, Porto-Novo, Benin; 4 International Fertilizer Development Center (IFDC), Camp Guezo, Lot 0134 Zone Résidentielle, 04 BP 673, Cotonou, Benin

**Keywords:** Computational biology and bioinformatics, Mathematics and computing

## Abstract

This study empirically tested the robustness of Bayesian Maximum Entropy (BME) in predicting spatiotemporal data, with an emphasis on skewness, sample size, and spatial dependency level. Simulated data, both Gaussian and non-Gaussian, were generated using the unconditional sequential simulation method, with sample sizes ranging from 100 to 500 at the interval length of 50 and varying skewness (0, 1, 3, 6 and 9) and spatial dependency levels (weak, moderate, and strong). Findings revealed sample size variations and spatial dependence levels did not significantly influence BME prediction’s Mean Square Error (MSE) and bias. While skewness significantly impacted MSE (p-value < 0.001), bias remained unaffected. Moreover, skewness and spatial dependence interactions affected both MSE and bias. Despite this, BME proved robust to sample size and skewness, demonstrating a negligible MSE on the graphical plot (heatmap).

## Introduction

Bayesian methodologies, particularly Bayesian Maximum Entropy (BME), have revolutionized probabilistic modeling by providing robust frameworks for complex stochastic systems^1^. BME represents a significant advance in geostatistical modeling, excelling in modeling spatial and spatio-temporal information, integrating diverse knowledge bases and managing accurate and uncertain data^2,7^. Several studies highlight the superiority of BME over traditional methods like kriging, Inverse distance weighted (IDW)^3,4^. BME uses soft and hard data, which refers to data with less precision and higher accuracy respectively^5^. Its accuracy is mainly enhanced by the use of soft data, improving prediction accuracy by 30% in modelling Escherichia coli concentrations^6^ and by 35.28% in Urban Climate Research, while also reducing field sampling cost^6^.

Geostatistical prediction involves estimating attributes of variables at unsampled locations^8^. The method is increasingly applied in many fields of science, including environmental sciences, soil sciences, ecology, remote sensing and public health^9^. Successful application of BME in soil sciences and related field, includes, rapid evaluation in earthquake analysis^10^; climate change impact assessment^11^; human disease modelling^12,13^ Soil mapping^14^, etc. This approach has proven invaluable in IoT applications for imputing missing sensor data^15^ and in handling skewed distributions without data transformation^16^.

In soil sciences, BME has been successfully applied by several authors^17–19^. However, the planning of any soil survey requires a sampling plan which specifies sample size and sampling grid^20^. In most spatial prediction methods, smaller grid sizes and higher resolutions can increase the accuracy and precision of spatial predictions^13^. The larger the sample size, the more significant the reduction of MSE obtained using the BME analysis method^22^. Several suggestions were made regarding sampling intensity for geostatistical analysis. Thus, Webster and Oliver^20^ proposed a minimum sample size of 100 locations, while they also suggested an optimum of 150 to 200 locations for a better variogram calculation. For soil analysis on a national scale, Sun^14^ recommended an optimal sample size to range from 500 to 1000 locations. However, most of the suggestions were made about the classical Geostatistics. In meta-analysis, Bayesian methods offer superior solutions for hierarchical data structures^23^, while advancements in computational power have expanded their application to economic modeling^24^. Therefore, it is necessary to numerically evaluate the robustness of BME to sample sizes and skewness. Specifically, we intend to numerically find out how robust is BME to skewness with varying sample sizes and spatial dependence levels.

BME technique was described as a robust method that is not sensitive to skewed and biased data, and can generate a posterior probability with minimum uncertainty from prior information^25^. Bayesian approaches address simulation study challenges such as uncertainty quantification and sensitivity analysis^26^, making them particularly effective in solving stochastic partial differential equations in complex simulations^27^. However, its predominant application has been on Gaussian probability distribution functions (pdfs)—typically associated with non-skewed data^22^. This creates a challenge when the data exhibits strong positive skewness, as often seen in environmental and soil data^28^. In such cases, a logarithmic transformation is applied to correct the skewness before using the BME method. Then, the posterior probability derived from the transformed variable is transformed back to yield the estimates of the variable^29^. Using simulated data, Orthon and Lark^22^ demonstrated that applying the standard BME method with logarithmic statistical moments included in the general knowledge base, gives a better result by reducing the MSE and bias of the prediction. The choice of BME for specific simulation studies is justified by its robustness, flexibility, and superior accuracy in complex, uncertain data environments, offering a comprehensive probabilistic description of systems under investigation.

In BME analysis, non-Gaussian pdfs arise from several sources, which result mainly from either the prior or the posterior stages. At the prior stage, non-Gaussian pdfs can be due to experimental data from moments of order higher than two or directly from the physical laws that govern the physical process. In contrast, at the posterior stage, a non-Gaussian posterior pdf can also result from a non-Gaussian prior pdf^30^. Also, environmental data, including soil data, often exhibit strong positive skewness. Therefore, the Gaussian assumption for the Spatial random field (SRF), may not be justified^31^. The skewed pdfs affect entropy measures and yield a smaller measure of entropy than a flatter pdf when a pdf has a greater degree of peakedness. A flat pdf shows a larger uncertainty about the variables thus, much information about this variable, while high peakedness shows more certainty, thus providing less information^22^. Also, when the data is strongly skewed, the derived variogram underestimates the spatial dependence due to a few tremendous values contributing to many squared differences^32^. Both sample size and sampling grids play a significant role in sampling natural resources. The grid and sample size contribution to prediction accuracy, is an important parameter in any environmental modelling. It is crucial for better monitoring and sound decision-making. In this study, we evaluated the impact of hard data skewness, sample size and spatial dependence on BME performance.

## Methods

Christakos^33^ introduced the concept of Bayesian Maximum Entropy (BME) as a product of epistemic reasoning in the study of natural phenomena. He explained that the distribution of a natural process over time and space is captured by two key knowledge bases: the General Knowledge Base (G) and the Site-Specific Knowledge Base. He further defined by (K) the sum of knowledge bases and expressed as:

1 $$K=G \cup S.$$

The General Knowledge Base encompasses a wide range of knowledge about a topic, including physical laws, scientific theories, and local laws. It is often estimated from the mean and the covariances of the variables of interest and expressed as:

2 $${\overline{ {\varvec{G}}} }_{\alpha }=\int d{X}_{{\rm map}} \, {\text{G}}_{{\varvec{\upalpha}}}({X}_{\text{map}}{)}{\mathcal{F}}_{{\varvec{G}}}{(}{X}_{\text{map}}{)}{ , }{\alpha }=1\dots ,{\text{N}}_{{\alpha }}$$

where ${X}_{\text{map}}\text{=}{\text{ [}{{X}_{data}}^{T}{{X}_{k}}^{T}] }^{T}$ includes all the data and estimation points, the data corresponding probability density function is ${\mathcal{F}}_{{\varvec{G}}}\text{(}{X}_{\text{map}}\text{)}$. ${\overline{G} }_{\alpha }$ is the space/time statistical moments of interest.

In contrast, the Specific Knowledge Base (S) refers to specific information organised into Hard data and soft data collected on a natural variable.

Assuming ${p}_{i}(i=1,\dots .,n)$ points location for a given natural situation in space and time. The site-specific knowledge could be expressed as:

3 $${ {\varvec{S}} :X}_{data}=({X}_{hard}{,X}_{soft}) =\text{ (}{X}_{1\dots \dots \dots \dots .}{X}_{n}).$$

Hard data are the exacts measures of the natural process ${{X}_{hard}\text{ = [}{X}_{1\dots \dots \dots \dots .}{X}_{{n}_{k}}] }^{T}$ at some locations ${p}_{i}(i=1,\dots .,{n}_{h})$. Hard data is sometime, the exact measurements of variable obtained from real observation devices, computational algorithms, or simulation processes^33,34^.

Soft data is uncertain observations expressed in terms of interval values, probability statements, empirical charts, assessments by experts, etc.^33,34^. It can be expressed as:

4 $${{X}_{soft}\text{ = [}{X}_{{n}_{k+1}}{\dots \dots .X}_{n}] }^{T} \text{at point }{p}_{i} \left(i={n}_{h+1},\dots .,n\right)$$

In practice, BME computation involves three basic steps namely the prior stage, the meta-prior stage and the posterior stage^35–37^^.^

### Prior stage

At this stage, data on general knowledge is collected and processed to build the prior distribution. The prior G pdf ${f}_{g}\left({x}_{map}\right)$. is derived with the constraints:

5 $${ Prob}_{G}\left[{X}_{\text{map}}\right]=p \epsilon \left[\text{0,1}\right]$$

### Meta-prior stage

In this step, the hard and soft data on the site are collected, arranged and transformed as possible to build the site-specific knowledge

6 $$\begin{array}{cc}& S: {x}_{\text{data }}=\left({x}_{\text{hard }},{x}_{\text{soft }}\right)=\left({x}_{1},{x}_{2},\dots ,{x}_{mh},{x}_{(mh+1)},\dots ,{x}_{m}\right) \\ & \text{ here }{x}_{\text{hard }}=\left({x}_{1},{x}_{2},\dots ,{x}_{mh}\right);{x}_{soft}=\left({x}_{(mh+1)},{x}_{(mh+2)},\dots ,{x}_{m}\right)\end{array}$$

### Posterior stage

In this step, information from Prior and Metaprior are tied together using logical rules. Therefore, the posterior step uses the laws of probability to process the available site-specific data using the prior pdf and considering soft and hard data, the Bayes condition is applied to ${f}_{g}\left({x}_{map}\right)$ to find the general posterior probability distribution function, ${f}_{k}\left({x}_{k}\right)$, expressed as follow:

7 $${f}_{k}\left({x}_{k}\right)={f}_{g}\left({x}_{k}\mid {x}_{d\varpi a}\right)=\frac{{f}_{g}\left({x}_{k},{x}_{\text{data }}\right)}{{f}_{g}\left({x}_{\text{data }}\right)}$$

In this research, BME robustness to spatial prediction was evaluated by testing several sample sizes, skewness’s and spatial dependence levels. The sample sizes considered were 100, 150, 200, 250, 300, 350, 400,450 and 500. Symmetric data (skewness = 0) and data positively skewed by 1, 3,6 and 9 were considered. Data with strong (≤ 0.25), moderate (0.25–0.77) and weak (< 0.75) relative nugget effects (NE)^38^^.^

8 $$NE=\frac{{c}_{0}}{{c}_{0}+c}$$

c: is the "sill" of a variogram which is the value at which the variogram levels off, indicating the point of maximum spatial variability that can be attributed to spatial correlation. In simple terms, the sill is a measure of the total variance (or the sum of the structured and random variance) in the dataset. When the variogram reaches the sill, it implies that beyond this point, the spatial correlation between data points no longer increases. While c_0​_ (Nugget), represents the variance at zero distance. It includes measurement error and micro-scale variation that are not captured in the spatial scale of the study. It effect can be thought of as the amount of variability that is not explained by spatial correlation. A high nugget value relative to the sill indicates a lot of random noise or variability that cannot be accounted for by spatial relationships.

Each spatial dependence level and the ranges were repeated three times, as shown in Table 1.

**Table 1:** Parameters considered in the simulation.

Spatial dependence	Scenarios	Nugget	Psill	Range
Strong	1	0.9	0.1	6
	2	0.8	0.2	5
	3	0.7	0.3	4
Moderate	1	0.5	0.5	6
	2	0.4	0.6	5
	3	0.3	0.7	4
Weak	1	0.2	0.8	6
	2	0.1	0.9	5
	3	0.05	0.95	4

### Data simulation

**Fig. 1:**
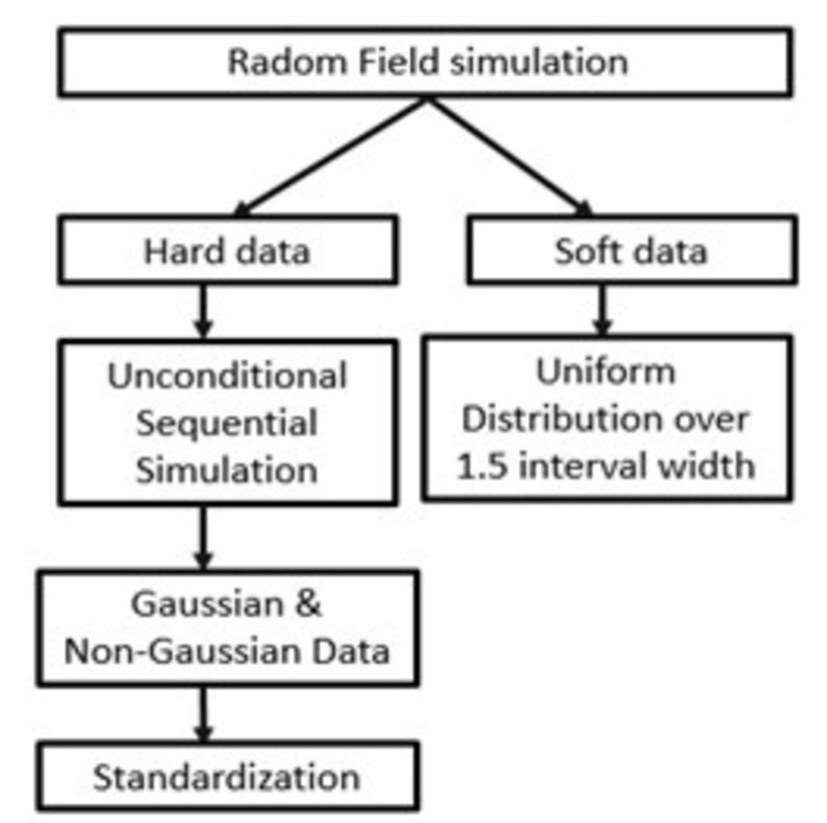
Flow chart of data simulation.

A random space field was simulated with an exponential covariance model having a sill *c*_0_+*c* of 1 and ranges over three spatial dependence levels (see Table 1)^22,39^. Two types of data were generated: hard and soft data. The hard data were generated using the unconditional sequential simulation method to generate gaussian and non-gaussian data^40,41^. For each spatial dependence level, fifteen thousand independent realizations of the standard gaussian variable were generated using a function built in R 4.3.2 (R Core Team 2019). A constant (range) was added to the value to yield a positive minimum value. Then, each value was raised to a given power, this exponentiation gives the data a positive coefficient of skewness. The data were then standardized to zero mean and unit variance^39^. The simulation function was based on the following arguments: Alpha (the exponentiation power to generate highly skewed values) and Skewness (the variable with this skewness is returned out of the 50,000 variables simulated: Sample size, Nugget effect, Effective range, Partial sill, and Variogram model). Interval-type soft data was assumed to be randomly located over the unit size square. The width of the intervals was equal to 1.5, such that the simulated values were uniformly distributed over the intervals. The simulation steps are summarised in the flowchart below (Fig. 1)

### Data analysis

The sample sizes vary from 100 to 500, so the largest sample size was considered a reference sample. The accuracy of each sample size (from 100 to 450) is compared to the reference sample (500) to find the optimum sample size. BME computation was performed with the library BMElib 2.0c^42^ using BME modes with the aim of predicting the best values at the nodes of grids.. Then, firstly, the exploratory analysis was applied to assess the overall data variability in estimates and the MSE summaries^43^. The bias of prediction was also estimated. Secondly, the effect of sample size, skewness and spatial dependence level were graphically plotted using R 4.3.2 (R Core Team, 2023). Finally, the significance of the effect of the factors considered (skewness, sample size, and spatial dependence) on the MSE and bias were assessed using Analysis of Variance (ANOVA).

## Results

### Effect of skewness, sample size and spatial dependence on BME performance

Mean Square Error (MSE) and bias are significantly affected by the interaction between the Skewness and the spatial dependence (SD), with p-value, respectively 0.016 and 0.019 (Table 2). However, the sample size, and its interaction with the spatial dependence and skewness do not affect BME accuracy. This indicates that BME is robust to sample size variation and spatial dependence but sensitive to the combined effect of skewness and spatial dependence.

**Table 2:** Test of sample size, skewness and spatial dependence on MSE and bias of BME prediction: results from the ANOVA.

Source of variations		MSE		Bias	
	DF	F – value	*Pr* (> *F*)	F-value	*Pr*(> *F*)
Skewness (SK)	4	116.94	< 0.001	1.91	0.1110
SD	2	1.23	0.293	2.73	0.067
Sample size (Ss)	7	0.38	0.912	0.28	0.962
SK:SD	8	2.40	0.016	2.36	0.019
SK:Ss	28	0.64	0.921	0.97	0.514
SD:Ss	14	0.85	0.617	0.64	0.829
Sk:SD:Ss	56	0.89	0.689	1.19	0.185

**Fig. 2:**
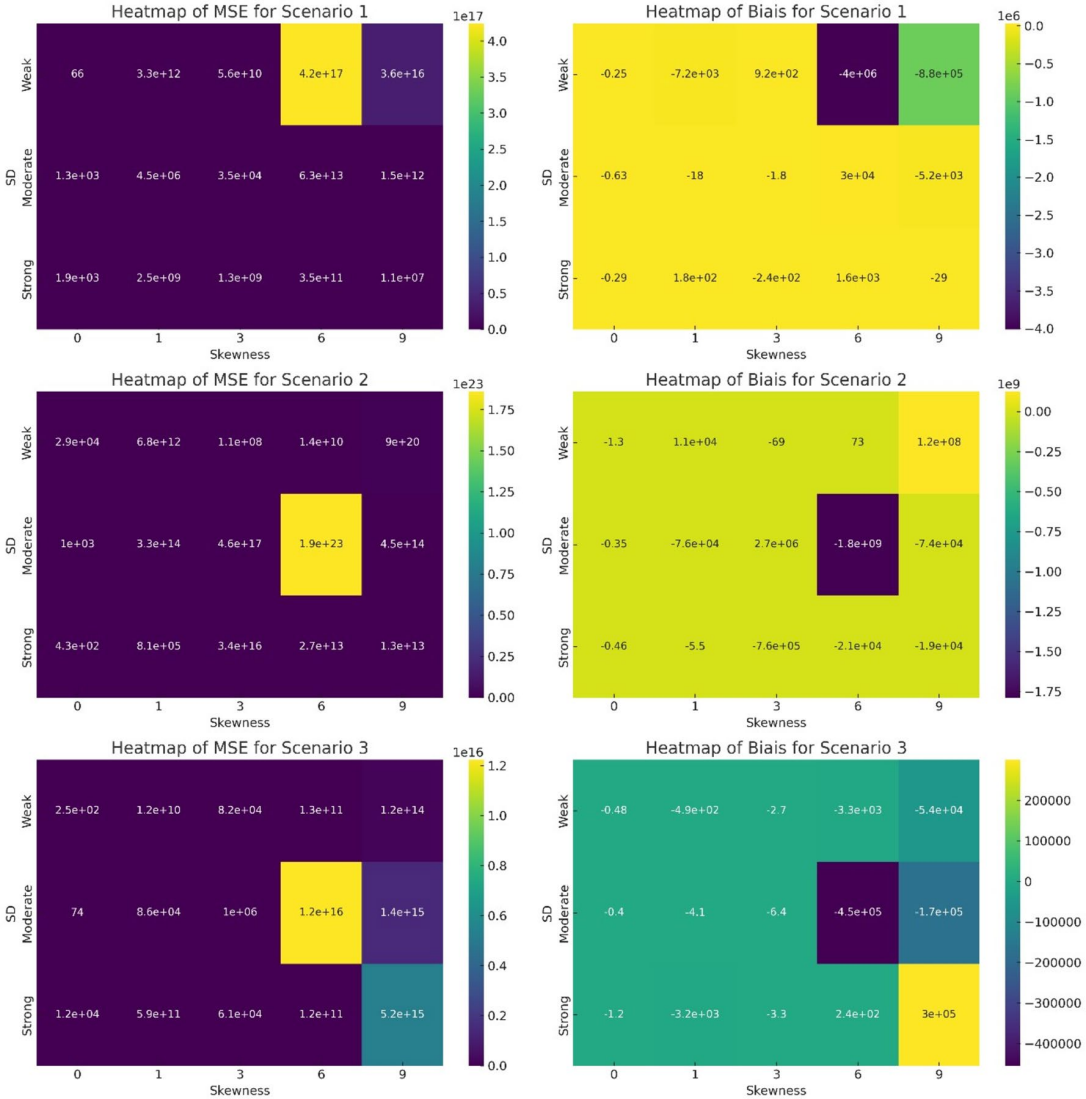
Heatmap of the interaction effect of Skewness and Spatial dependence (SD) on MSE and Bias of prediction.

In the heatmap analysis (Fig. 2), a consistent pattern emerged across the three scenarios, indicating a uniform response to varying levels of Skewness and Spatial Dependence (SD). Notably, we observed that when skewness is below 6, the level of SD (Weak, Moderate, Strong) does not significantly impact the Mean Squared Error (MSE) and Bias of the predictions. This suggests that for lower skewness values, the predictive model's accuracy and bias are relatively unaffected by the degree of spatial dependence.

However, a critical threshold is observed at a skewness level of 6 and above. Beyond this point, the SD begins to significantly influence the model's performance. Higher skewness levels, coupled with varying SD, lead to noticeable changes in both MSE and Bias. This shift highlights the increased sensitivity of the predictive accuracy to spatial dependence under conditions of higher skewness. The uniformity of this trend across all scenarios underscores the robustness of this observation and its potential implications for predictive modelling in similar data environments.

## Discussion

Bayesian Maximum Entropy is widely used nowadays in spatiotemporal mapping and is presented as more reliable in estimation^36^,^44^. However, Gongnet et *al*.^28^ carried out a review on the use of sample size, skewness and spatial dependence. His result pointed out a low level of awareness on their use. The use of small sample sizes leading to non-optimal variogram and the application of BME on both the transformed and untransformed variable when data is highly skewed. This research was carried out to provide better understanding on BME robustness in spatial estimation. Our result shows the influence of skewness and its interaction with spatial dependence on the accuracy of Bayesian Maximum Entropy (BME) predictions, as shown in previous studies^33^,^45^. The skewness significantly affected the MSE of prediction as well as the interaction with the spatial dependence. In particular, skewness values of 6 and 9 under strong spatial dependence conditions reveal a marked impact on prediction accuracy. Moderate spatial dependence exhibits significant differences only for skewness of 9^46^. Weak spatial dependency, however, shows significant differences from 0 for skewness values of 3, 6, and 9^47^. However, BME can be extended to handle non-Gaussian or skewed data by incorporating a transformation of the data or by using non-Gaussian priors^48,49^. This allows BME to provide accurate and robust predictions for datasets with a variety of distributions, including those with heavy tails or skewness. Despite the significant differences due to skewness, a graphical plot reveals values of MSE close to zero, suggesting that BME can be considered robust to skewness. Also, more robust in handling skewed data than other methods such as Kriging that are less capable of dealing with non-Gaussian data^48,49^. This is consistent with the review of^22^, who stated that BME’s perform well for both Gaussian and non-Gaussian data. Our findings align also with Banerjee et al.^50^ and the recent analysis of BME prediction bias, highlighting that skewness values exceeding 3 significantly impact both MSE and bias, regardless of spatial dependency. The interaction between skewness and spatial dependence, particularly in strong spatial dependence scenarios, enhances prediction accuracy with higher skewness values^51^. This insight is crucial for fields where data frequently exhibit strong spatial dependencies, such as geospatial studies or environmental science. This study revealed that MSE and bias of prediction is not affected by sample size. This aligns with the works Christakos who presented BME as robust to varying sample size resulting from the incorporation of prior knowledge about the spatial distribution in the form of a covariance function or a variogram^33^. This allows the method to make accurate predictions even in cases where the sample size is small. It makes BME more powerful in estimation compared to other methods such as kriging, which may be more sensitive to sample size^40^. Our results underscore the need to consider data distribution and inherent spatial structures in predictive modeling^39,43^. Future research should continue to explore these factors to refine BME prediction accuracy.

## Conclusion

In conclusion, our study affirms the significant role of skewness and spatial dependence in BME prediction accuracy and bias. Although the MSE values are close to zero, suggesting robustness to skewness, the interplay between skewness and spatial dependence remains a crucial consideration for enhancing the performance of BME models. Therefore, BME can be considered robust to sample size, spatial dependence and skewness (less than 6). Overall, these results underscore BME's suitability for handling diverse data distributions and complexities in spatial modeling, reaffirming its superiority over traditional methods like kriging. The study's insights are valuable for fields reliant on accurate spatial predictions, such as environmental sciences and geostatistics, providing a nuanced understanding of BME's robustness and limitations in practical applications.

## Data Availability

The datasets used in this study was simulated and are available from the corresponding author on request.
